# Supplementary material for: Functional and Phenotypic Characterization of Siglec-6 on Human Mast Cells
Source: Cells. 2022 Mar 28;11(7):1138. doi: 10.3390/cells11071138 (PMC8997871; doi:10.3390/cells11071138)
Supplement: Supplementary file 1 [file cells-11-01138-s001.zip › cells-1639535-supplementary.pdf]

Functional and phenotypic characterization of Siglec-6 on human mast cells

Supplemental Information

Materials and Methods

Patient Recruitment

Pediatric patients with suspected or established diagnosis of eosinophilic esophagitis were recruited for collection of esophageal biopsies for single cell RNA-Sequencing. Informed consent was obtained from two parents and assent for patients 12 years old and over prior to obtaining research specimens. Active EoE was diagnosed based on the presence of symptoms of esophageal dysfunction and 1 or more biopsies showing a peak eosinophil count of at least 15 eosinophils per high-powered-field (eos/hpf) determined by a pathologist. Patients with an established diagnosis of EoE were considered inactive EoE if all biopsies examined by a pathologist showed less than 15 eos/hpf. Non-EoE controls were patients with symptoms of esophageal dysfunction and less than 15 eos/hpf on a diagnostic endoscopy. Treatments for the EoE patients included diet elimination, swallowed steroids, or proton pump inhibitors. This work was conducted under IRB #2011-14486, which was approved by Ann & Robert H. Lurie Children's Hospital of Chicago. The final cohort included 7 patients with active EoE, 4 with inactive EoE, and 1 non-EoE control.

Single cell isolation

Two esophageal tissue biopsies were collected at the time of standard-of-care endoscopy in transfer buffer (RPMI 1640, 10% Fetal Bovine Serum [FBS], 1% Penicillin-Streptomycin [all from ThermoFisher Scientific, Waltham, MA]) on ice and transferred to the laboratory within 30 minutes. The tissue biopsy was removed from the transfer buffer and washed with cold wash buffer (Hanks balanced salt solution [HBSS], 20 mM N-2-Hydroxyethylpiperazine-N'-2-Ethanesulfonic Acid [HEPES] [all from ThermoFisher Scientific, Waltham, MA]) by gently inverting the tube several times. The tissue was incubated with preheated EDTA stripping buffer (Hanks balanced salt solution [HBSS], 5mM Ethylenediaminetetraacetic acid [EDTA], 10mM HEPES, 10mM Dithiothreitol (Sigma-Aldrich, St. Louis, MO), 10% FBS) thrice for 10 mins at 37°C to loosen the tissue, and then washed thrice with cold wash buffer to get rid of dead cells/debris. The sample was minced with a pair of scissors and digested in 1ml of preheated Collagenase digestion buffer (2.5mg Roche Collagenase A (Sigma-Aldrich, St. Louis, MO), RPMI-1640, 10% FBS) at 37°C for 20 minutes in a thermomixer at a speed of 750 rpm. The cell mixture was passed through a 19G syringe (BD PrecisionGlide Needle) and filtered through a 30μM MACS strainer (Miltenyi Biotec, Cambridge, MA) into a 15mL conical by repeatedly rinsing the syringe and strainer with RPMI/10%FBS wash buffer until the final volume of the cell suspension was 15mL. The cells were pelleted on centrifuge at 1200rpm for 5mins at 4°C and

the supernatant was aspirated. The cell pellet was resuspended in 100µL of cold PBS/1% BSA buffer, and the cell concentration and viability were analyzed with a Nexcelom Cellometer with Acridine Orange/Propidium Iodide (AOPI).

Single-cell library preparation and sequencing

Sixteen thousand cells were loaded into the Chromium Controller (10X Genomics, PN-120223) on a Chromium Next GEM Chip G Single Cell Kit for v3.1 Chemistry (10X Genomics, PN-1000120); and processed to generate single cell gel beads in the emulsion (GEM) according to the manufacturer's protocol. The cDNA libraries were generated using the Chromium Next GEM Single Cell 3' Reagent Kits v3.1 (10X Genomics, PN-1000121) according to the manufacturer's manual. The quality of the constructed library was analyzed with Agilent Bioanalyzer High Sensitivity DNA kit (Agilent Technologies, 5067-4626) and Qubit DNA HS assay kit for qualitative and quantitative analysis, respectively. The multiplexed libraries were sequenced on an Illumina Hiseq 4000 with 100 cycle kit using the following read length: 28 bp Read1 for cell barcode and UMI, and 90 bp Read2 for transcript. The targeted sequencing depth for gene expression were 20,000 reads per cell.

Bioinformatics analysis

Raw sequencing read data was analyzed with FastQC to assess quality and aligned to the hg38 human reference genome using Cellranger (v3). Sparse data matrices were used as input into Seurat (v4.0) analyzed in R (v3.0). For analysis of all sequenced samples, cells with less than 250 unique genes, 250 UMI counts, and less than 0.7 of log10GenesPerUMI were filtered, along with genes with zero counts in all cells. Ambient RNA contamination was removed for each sample individually using decontX function (celda v1.8.1), and the samples were merged into a single object for analysis resulting in 44,153 cells and 24,068 genes. The gene expression was log-normalized and variable genes were identified with the FindVariableFeatures function. The data was scaled with regression for the number of UMI and percentage of mitochondrial genes in each cell. Principal component analysis (PCA) was next performed, and the top 10 components were used to generate a Uniform Manifold Approximation and Projection (UMAP). The data was subjected to shared nearest neighbor modularity optimization-based clustering, with the aid of an elbow plot using 30 dimensions and a resolution of 0.1, which identified 13 clusters. The top expressed gene markers for each cluster were determined, and clusters were annotated with the canonical markers to identify the following cell types: Mast cells, Myeloid cells, T-cells, Fibroblasts, Endothelial cells, and Epithelial cells. Cell identities were confirmed using SingleR (v1.8.1), which identified B-cells as part of the myeloid cell cluster and were further annotated. Dot plots were used to visualize the relative expression of top genes with high specificity for each cell type. In total, 1753 mast cells were identified. Violin plots were used to visualize the relative expression of the SIGLEC6 and SIGLEC8 genes among each cell types for each disease state: active EoE, inactive EoE, and non-EoE control.

Secondary antibody co-crosslinking of FcγRII and Siglec-6 on THP-1 cells

THP-1 cells were incubated with or without anti-hFcγRII (clone IV.3; Stemcell Labs, Cambridge, MA) at 5 μg/ml, anti-Siglec-6 at 10 μg/ml, and goat anti-mouse IgG $F(ab')_2$ (Jackson ImmunoResearch, West Grove, PA) at 50 μg/ml at 4° for 30 min prior to washing and incubating the cells for 5 min at 37°C to permit cell stimulation. Activating signaling through FcγRII was evaluated by total intracellular phosphotyrosine levels by flow cytometry.

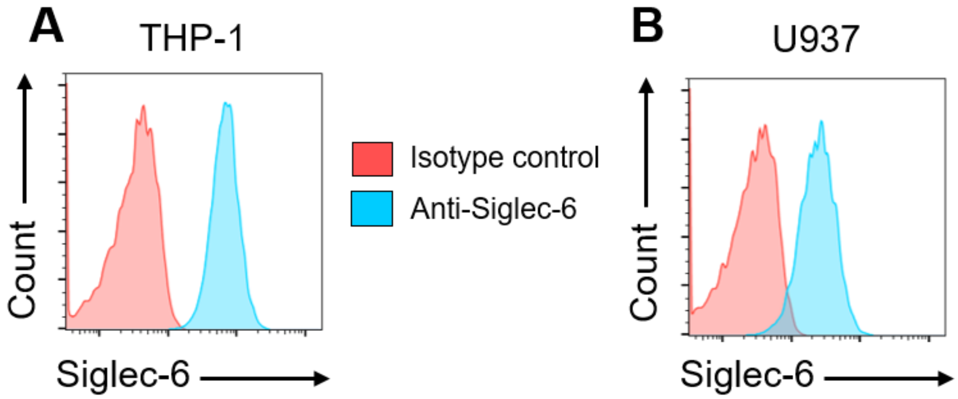

Figure S1. Cell-surface Siglec-6 expression on acute myeloid leukemia cell lines THP-1 and U937. Cell-surface Siglec-6 expression was determined by anti-Siglec-6 mAb binding by flow cytometry on THP-1 (A) and U937 (B) cells relative to binding of an isotype control mAb. Data are representative of three independent experiments.

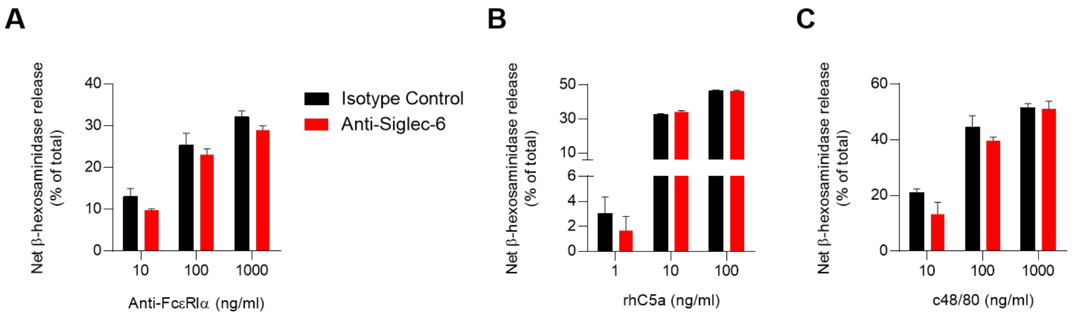

Figure S2. Siglec-6 antibody ligation reduces β-hexosaminidase release. HSMCs were collected, washed, and incubated in the presence or absence of anti-Siglec-6 mAb for 30 min. HSMCs were then incubated with the indicated concentrations of anti-FcεRI mAb **(A)**, rhC5a **(B)**, or compound 48/80 **(C)** for 30 min. Release of β-hexosaminidase was assessed by colorimetric assay in the supernatant, and net β-hexosaminidase release was determined by calculating the percent released relative to the total (amount in supernatant and lysate) and subtracting the baseline β-hexosaminidase release in the absence of stimulation (2.53116% [A], 5.20354% [B], and 5.40341% [C]). Data represent the means and standard deviations of 3 replicates and are representative of results from 5 (A) or 4 (B,C) distinct HSMC cultures.

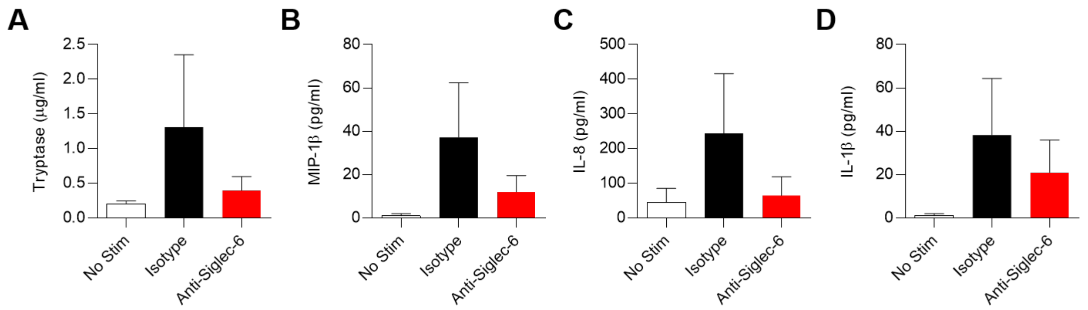

Figure S3. Co-crosslinking of Siglec-6 and FcεRIα on MCs reduces levels of released tryptase, MIP-1β, IL-8, and IL-1β. CD34+ cell-derived MCs were incubated with 150 ng/ml anti-FcεRIα and either anti-Siglec-6 or isotype control mAb or were not incubated with mAbs (No Stim). Antibodies were crosslinked using secondary anti-mouse IgG antibody. Tryptase release **(A)** was measured colorimetrically in cell-free supernatant after 20 min of stimulation. MIP-1β **(B)**, IL-8 **(C)**, and IL-1β **(D)** were detected in cell-free supernatant after overnight stimulation. Data represent the means and standard deviations of 4 (A,B) or 3 (C,D) independent mast cell cultures and experiments.

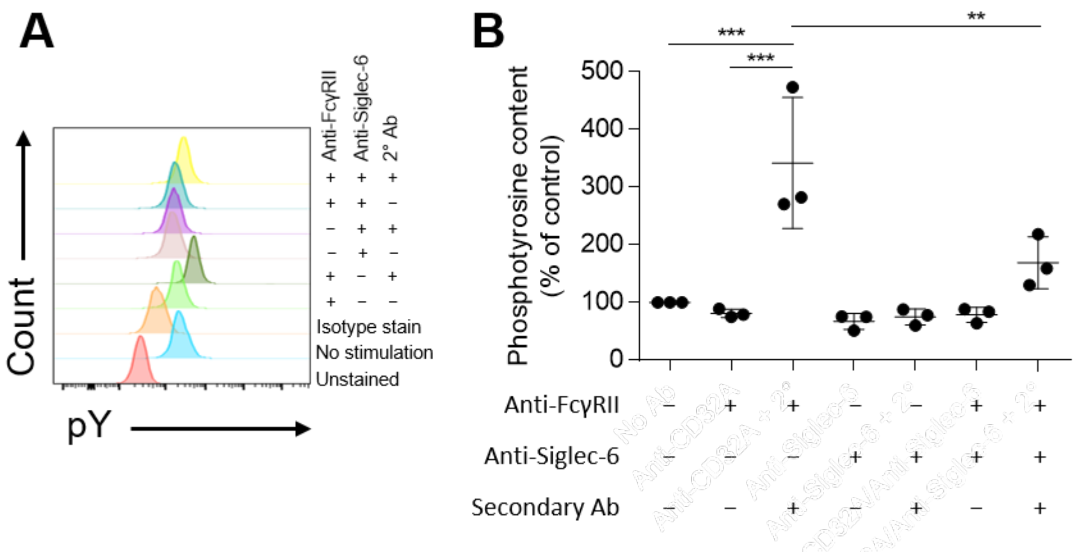

Figure S4. Co-aggregation of Siglec-6 and FcγRII inhibits activating protein tyrosine kinase activity in THP-1 cells. THP-1 cells were incubated with the indicated combination of anti-FcγRII, anti-Siglec-6, and secondary cross-linking antibody (2° Ab) for 5 min at 37° prior to fixation and permeabilization. Levels of cytoplasmic phosphotyrosine were then determined by intracellular staining and flow cytometry. (A) Histograms demonstrate intracellular phosphotyrosine (pY) staining for each antibody treatment and staining control. (B) Phosphotyrosine content was quantified by subtracting the MFI of the isotype control mAb-stained sample from the experimental samples and normalizing to the untreated control sample (No stimulation). Data are representative (A) or represent the individual values, means, and standard deviations of three independent experiments (B). **, $p < 0.01$; ***, $p < 0.001$ by one-way ANOVA with Tukey test to correct for multiple comparisons.
